# Complete Genome Sequence of Lactobacillus johnsonii NCK2677, Isolated from Mice

**DOI:** 10.1128/MRA.00918-20

**Published:** 2020-10-22

**Authors:** Sarah O'Flaherty, Matthew H. Foley, Alissa J. Rivera, Casey M. Theriot, Rodolphe Barrangou

**Affiliations:** a Department of Food, Bioprocessing, and Nutrition Sciences, North Carolina State University, Raleigh, North Carolina, USA; b College of Veterinary Medicine, Department of Population Health and Pathobiology, North Carolina State University, Raleigh, North Carolina, USA; University of Maryland School of Medicine

## Abstract

We report the closed genome sequence of a Lactobacillus johnsonii strain (NCK2677) that was isolated from a cefoperazone-treated mouse model designed for the study of Clostridioides difficile infection. Illumina and Nanopore sequencing reads were assembled into a circular 1,951,416-bp chromosome with a G+C content of 34.7%, containing 1,865 genes.

## ANNOUNCEMENT

Lactobacilli, including Lactobacillus johnsonii, are considered beneficial bacteria with applications as probiotic and therapeutic bacteria in both humans and animals. We report the complete genome sequence of Lactobacillus johnsonii NCK2677, which was isolated from a cefoperazone-treated mouse model designed for the study of Clostridioides difficile infection (1, 2).

Fecal samples obtained from wild-type C57BL/6 mice (The Jackson Laboratory, Bar Harbor, ME) after cefoperazone treatment were resuspended in phosphate-buffered saline and plated onto *Lactobacillus* selection (LBS) agar to select individual colonies. Isolated colonies were then inoculated into MRS broth, grown overnight at 37°C under ambient atmospheric conditions, and restreaked twice onto MRS agar plates to confirm purity. L. johnsonii colonies were confirmed by amplification of the variable region of the 16S rRNA gene using the primer pair plb16 (5′-AGAGTTTGATCCTGGCTCAG-3′) and mlb16 (5′-GGCTGCTGGCACGTAGTTAG-3′), as described by Kullen et al. (3). The resulting amplicon was sequenced by Sanger sequencing (Genewiz, Durham, NC), and DNA sequences were used to confirm the species identity with a BLAST search against publicly available sequences in the NCBI database. One confirmed L. johnsonii colony was inoculated into MRS broth and grown overnight, and 15 ml of the culture was centrifuged at 1,717 × *g* for 10 min. The cell culture pellet was stored at −80°C. DNA extraction, library preparation, whole-genome sequencing, and assembly were performed at the Roy J. Carver Biotechnology Center (University of Illinois at Urbana-Champaign, Urbana, IL). Genomic DNA was extracted with the MasterPure DNA purification kit (Lucigen). For DNA sequencing with the Illumina platform, shotgun genomic libraries were prepared with the KAPA HyperPrep library construction kit (Roche). The libraries were quantitated by quantitative PCR and sequenced in one lane for 251 cycles, from each end of the fragments, on a HiSeq 2500 system using the HiSeq Rapid SBS sequencing kit v2. Fastq files were generated and demultiplexed with bcl2fastq v2.20 conversion software (Illumina). The genomic DNAs were converted into Nanopore libraries with the NBD114 and 1D (SQK-LSK109) library kits. The libraries were sequenced in two SpotON R9.4.1 FLO-MIN106 flow cells for 72 h, using a GridION X5 sequencer. Base calling was carried out using Guppy v3.0.3, and adaptors were removed with Porechop v0.2.3 (Nanopore). The Nanopore sequencing generated 201,347 long reads (974,430,705 bp) with an *N*_50_ value of 6.3 kb. The Illumina sequencing generated 1,274,082 read pairs (2 × 250 bp).

To assemble the genome, Illumina reads and Nanopore long reads were checked for quality prior to and after trimming using FastQC v0.11.8 (http://www.bioinformatics.babraham.ac.uk/projects/fastqc). Default parameters were used for all software unless otherwise specified. Illumina reads were trimmed using Trimmomatic v0.36 (4) with parameters set ILLUMINACLIP:TruSeq3-PE-2.fa:2:15:10 LEADING:28 TRAILING:28 MINLEN:30; this retained reads longer than 30 bp and resulted in 1,268,909 read pairs. Long reads were adapter trimmed with Porechop v0.2.3 (https://github.com/rrwick/Porechop) and length filtered to a minimum of 1 kb with seqtk v1.2 (https://github.com/lh3/seqtk). Reads were assembled with Unicycler v0.4.4 (5).

Unicycler assembled the trimmed Illumina reads and uncorrected Nanopore reads in a hybrid assembly using the default normal mode. Within Unicycler, the Illumina reads were assembled with SPAdes v3.11.1 (6), and the resulting long-anchor contigs were assembled together with the Nanopore reads with an optimized version of miniasm (7) and Racon v0.5.0 (8). Pilon v1.22 (9) was used within Unicycler to iteratively polish the assembly with the Illumina reads. The circularized genome was rotated to the default starting gene *dnaA*, resulting in a 1,951,416-bp chromosome with a G+C content of 34.7%. The NCBI Prokaryotic Genome Annotation Pipeline (PGAP) v4.8 was used for annotation (10, 11) and identified 1,865 genes, including 1,762 protein-encoding genes and 79 tRNAs.

### Data availability.

This sequencing project has been deposited in GenBank under accession number CP059055. The associated BioProject and BioSample accession numbers are PRJNA645941 and SAMN15520417, respectively. The associated SRA accession numbers are SRR12296539 (Nanopore reads) and SRR12296540 (Illumina reads).
